# The 2A linker outperforms NAL10 in bicistronic translational efficiency for transgene stacking

**DOI:** 10.1093/hr/uhag170

**Published:** 2026-04-27

**Authors:** Yuan-Jie Deng, Rong-Rong Zhang, Ao-Qi Duan, Shan-Shan Tan, Shan-Shan Liu, Ai-Sheng Xiong

**Affiliations:** State Key Laboratory of Crop Genetics & Germplasm Enhancement and Utilization, Ministry of Agriculture and Rural Affairs Key Laboratory of Biology and Germplasm Enhancement of Horticultural Crops in East China, College of Horticulture, Nanjing Agricultural University, 1 Weigang, Nanjing 210095, China; State Key Laboratory of Crop Genetics & Germplasm Enhancement and Utilization, Ministry of Agriculture and Rural Affairs Key Laboratory of Biology and Germplasm Enhancement of Horticultural Crops in East China, College of Horticulture, Nanjing Agricultural University, 1 Weigang, Nanjing 210095, China; State Key Laboratory of Crop Genetics & Germplasm Enhancement and Utilization, Ministry of Agriculture and Rural Affairs Key Laboratory of Biology and Germplasm Enhancement of Horticultural Crops in East China, College of Horticulture, Nanjing Agricultural University, 1 Weigang, Nanjing 210095, China; State Key Laboratory of Crop Genetics & Germplasm Enhancement and Utilization, Ministry of Agriculture and Rural Affairs Key Laboratory of Biology and Germplasm Enhancement of Horticultural Crops in East China, College of Horticulture, Nanjing Agricultural University, 1 Weigang, Nanjing 210095, China

Dear Editor,

Transgene stacking is an important technique in plant synthetic biology, which allows simultaneous expression of multiple genes. The traditional methods of combinatorial transformation or hybridization between transgenic plants are time-consuming and inefficient, while transgene stacking is the preferred method for researchers. In transgene stacking process, promoter selection and cargo limit often restrict its application in synthetic biology. For example, multiple use of the same promoter led to the simultaneous silence of target genes. Also, ordinary vectors do not possess the huge cargo ability such as ten gene cassettes. Furthermore, multiple gene cassettes are challenging in vector construction. New tools are needed to solve this situation.

2A and NAL sequences were found to obtain the ability to translate two proteins as a polycistron [1, 2]. These two linkers were applied for generating carotenoid-biofortification crops [1]. Combining gene linkers with multiple gene cassettes strategy will greatly solve the problem of vector construction. Previously, a visible reporter, RUBY, was developed for monitoring plant transformation [3]. This reporter was an excellent tool for testing gene linkers’ efficiency. Also, expression of the reporter in horticultural plants could easily generate new-to-nature germplasms biofortified with betalain pigments.

Although 2A and NAL gene linkers have been reported, a direct comparison of their functional performance in applied plant synthetic biology contexts is lacking, thus hampering the selection strategies. In this study, we aim to compare the efficiency between 2A and NAL using two visible reporters.

We selected the F2A, which was used in the well-established RUBY reporter system and most efficient NAL10 linker in later experiments [1, 3]. F2A (CAATTGTTGAATTTTGATTTGTTGAAGTTGGCTGGAGATGTTGAATCTAATCCTGGACCT) and NAL10 (CAATCAAAC) were 60 and 9 bp long, respectively. First, we generated two constructs, RUBY-2A and RUBY-S-2A, in which stop codons of *BvCYP76AD1* and *BvDODA1* were removed (Fig. 1a and b). *Agrobacterium tumefaciens* (*A. tumefaciens*, GV3101) harboring RUBY-2A and RUBY-S-2A were infiltrated into *Nicotiana benthamiana* (*N. benthamiana*) leaves, respectively (Fig. 1c). As expected, *N. benthamiana* leaves expressing RUBY-2A and RUBY-S-2A all turning red due to the accumulation of betacyanins (Fig. 1c). F2A was efficient for stacking two or three transgenes and only expressing two genes, *BvCYP97AD1* and *BvDODA1*, were sufficient to induce betacyanins production in *N. benthamiana*. We constructed a RUBY-S-NAL in which *BvCYP97AD1* and *BvDODA1* was linked by the NAL10 linker (Fig. 1d). The stop codon of *BvCYP97AD1* was kept in RUBY-S-NAL. The presence or absence of the stop codon is dictated by the distinct operative mechanisms of the two linkers: ribosome-skipping for F2A and translation re-initiation for NAL10. *A. tumefaciens* harboring RUBY-S-NAL and RUBY-S-2A were then infiltrated into *N. benthamiana*. Four days after infiltration, the *N. benthamiana* accumulated betacyanins as expected but in different content. *N. benthamiana* expressing RUBY-S-NAL and RUBY-S-2A were pale red and deep red, respectively (Fig. 1e). The betacyanin content of RUBY-S-NAL was lower than RUBY-S-2A and was 16.6% of RUBY-S-2A (Fig. 1f). Expression levels of two betalains biosynthesis genes (*BvCYP76AD1* and *BvDODA1*) were determined by RT-qPCR (Fig. 1g). To avoid interference from plasmids in transient expression assays, all RNA preparations were rigorously treated with RNase-free DNase I to completely degrade any contaminating genomic or plasmid DNA. Expression levels of *BvCYP76AD1* and *BvDODA1* were nearly similar between RUBY-S-NAL and RUBY-S-2A samples.

**Figure 1:**
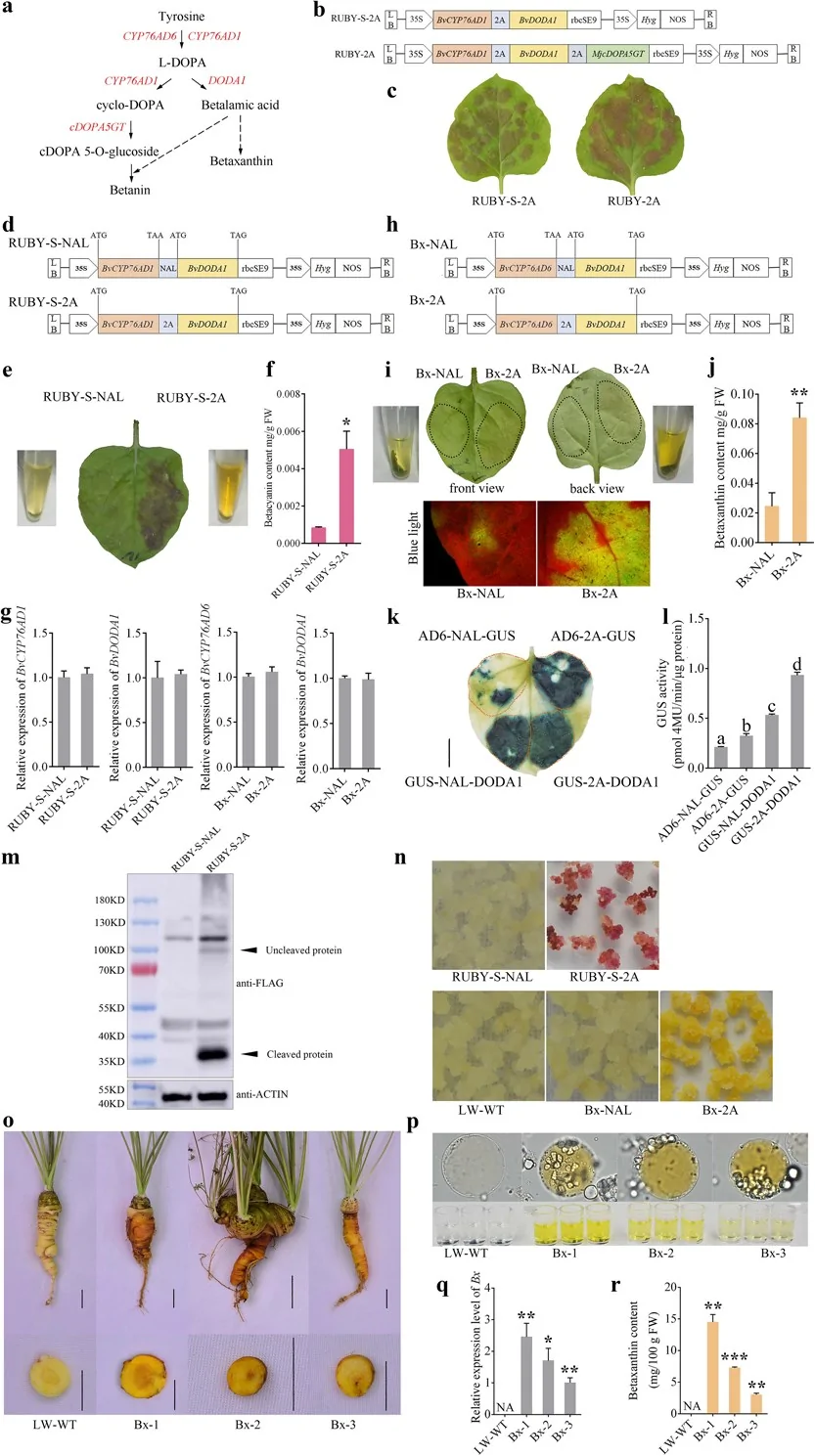
Efficiency comparison of 2A and NAL10 in transgene stacking. (a) Pathway of betalains biosynthesis in plants. Solid arrows show enzymatic reactions. Dotted arrows show spontaneous reactions. (b) Construct of the RUBY-S-2A and RUBY-2A. (c) Infiltration of RUBY-S-2A and RUBY-2A in *N. benthamiana*. (d) Construct of RUBY-S-NAL and RUBY-S-2A. (e) Infiltration of RUBY-S-NAL and RUBY-S-2A in *N. benthamiana*. (f) Betacyanin content in *N. benthamiana* expressing RUBY-S-NAL and RUBY-S-2A. Values are means ± SD from 8 biological replicates. (g) Relative expression level of betalain biosynthesis genes in transgenic *N. benthamiana*. *NtActin* was used as the reference gene. The relative expression level in the RUBY-S-NAL or Bx-NAL was set as 1. (h) Construct of Bx-NAL and Bx-2A. (i) Infiltration of Bx-NAL and Bx-2A in *N. benthamiana*. (j) Betaxanthin content in *N. benthamiana* expressing Bx-NAL and Bx-2A. Values are means ± SD from 8 biological replicates. (k) GUS staining of transgenic *N. benthamiana*. Scale bar = 1 cm. (l) GUS activity of transgenic *N. benthamiana*. Different letters indicate significant difference between samples using one-way ANOVA at *P* < 0.05 (*n* = 8 plants, ±SD). (m) Western blot analysis of BvDODA1 and fusion protein expression. (n) Carrot calli expressing RUBY-S-NAL, RUBY-S-2A, Bx-NAL and Bx-2A. (o) Phenotype of transgenic carrot taproots expressing Bx-2A. (p) Protoplast observation by light microscopy. (q) Relative expression levels of *Bx* in carrot taproots. *DcActin* was used as reference gene. The relative expression level of Bx-3 sample was set as 1. Values are means ± SD from 3 biological replicates. (r) Betaxanthin content in carrot taproots. Values are means ± SD from 3 biological replicates. Asterisks indicate significant difference in comparison with WT or control sample (^*^  *P* < 0.05; ^**^  *P* < 0.01, ^***^  *P* < 0.001 level, based on Student’s *t* test).

We also used betaxanthin reporter to check above results. *BvCYP76AD6* and *BvDODA1* were linked by F2A sequence and stop codon of *BvCYP76AD6* was removed resulting in Bx-2A construct (Fig. 1h). *BvCYP76AD6* and *BvDODA1* were also linked by NAL10 resulting in Bx-NAL construct, and stop codon of *BvCYP76AD6* was kept (Fig. 1h). *A. tumefaciens* harboring these constructs were infiltrated into *N. benthamiana*, respectively. Four days after infiltration, *N. benthamiana* expressing Bx-2A turned vivid yellow (Fig. 1i). However, *N. benthamiana* expressing Bx-NAL showed no obvious yellow patches (Fig. 1i). *N. benthamiana* expressing Bx-2A emitted visible yellow fluorescence under blue light due to the overlay of green and red fluorescence of betaxanthin and chlorophyll, respectively (Fig. 1i). *N. benthamiana* expressing Bx-NAL barely emitted green fluorescence and mostly red fluorescence was observed (Fig. 1i). Betaxanthin content was significantly lower in Bx-NAL than Bx-2A and was 29.1% of Bx-2A (Fig. 1j). Expression levels of two betalains biosynthesis genes were determined by qRT-PCR (Fig. 1g). Expression levels of *BvCYP76AD6* and *BvDODA1* were nearly similar between Bx-NAL and Bx-2A samples, respectively. The above results demonstrated that F2A was a more efficient gene linker than NAL10.

It seemed that transcription efficiency of mRNAs was similar between two linkers. We further attempted to compare efficiency of protein translation between two linkers. For this purpose, we used GUS as a reporter gene and replaced *BvCYP76AD6* and *BvDODA1* in Bx-NAL and Bx-2A constructs with GUS, respectively. Finally, four final constructs AD6-NAL-GUS (AD6 represented BvCYP76AD6), AD6-2A-GUS, GUS-NAL-DODA1(DODA1 represented BvDODA1) and GUS-2A-DODA1 were obtained. These four constructs were expressed in *N. benthamiana* individually, and GUS staining was performed (Fig. 1k). The infiltration regions of *N. benthamiana* expressing AD6-2A-GUS, GUS-NAL-DODA1, and GUS-2A-DODA1 were all dark blue (Fig. 1k). The infiltration regions of *N. benthamiana* expressing AD6-NAL-GUS showed spotted staining pattern and in most infiltrated regions no staining was observed (Fig. 1k). GUS activity was lowest in *N. benthamiana* expressing AD6-NAL-GUS, followed by AD6-2A-GUS, GUS-NAL-DODA1, and GUS-2A-DODA1 (Fig. 1l). Western blot analysis was performed to detect the protein levels of the two enzymes expressed from the RUBY-S-2A and RUBY-S-NAL constructs (Fig. 1m). A 3^*^FLAG tag was fused to the C-terminus of the BvDODA1 protein for detection. For the RUBY-S-2A construct, both the uncleaved fusion protein (~90 kDa) and the cleaved BvDODA1 product (~34 kDa) were detected. The cleaved BvDODA1 was the predominant form, indicating a high cleavage efficiency of 91.20% for the F2A peptide. For the RUBY-S-NAL construct, neither the full-length fusion protein nor the separate BvDODA1 protein was detectable via western blot. The vastly different efficiencies of the NAL (translation re-initiation) and F2A (ribosome skipping) mechanisms result in extremely low protein yield from the NAL construct—below the detection limit of standard Western blotting. However, the high catalytic activity and signal amplification inherent to betalain synthesis allow this minimal enzymatic activity to produce a faint yet detectable red color, explaining the apparent discrepancy between the functional phenotype and protein detection. These results indicated that efficiency of protein translation of NAL10-linked polycistrons (especially the second protein) were severely hindered.

Above four constructs (RUBY-S-NAL, RUBY-S-2A, Bx-NAL, and Bx-2A) were further transformed individually into carrot (cultivar ‘Lunar white’, LW) medicated by *A. tumefaciens* individually. Carrot calli expressing RUBY-S-2A and Bx-2A turned red and vivid yellow due to the accumulation of betacyanins and betaxanthins, respectively (Fig. 1n). However, carrot calli expressing RUBY-S-NAL or Bx-NAL appeared pale yellow like wild-type calli (note: more than 200 positive calli of each construct were obtained) (Fig. 1n). We previously had generated RUBY-S-2A transgenic carrot lines, which accumulated significant betacyanins. Three Bx-2A transgenic carrot lines were obtained and taproots all turned vivid yellow to orange compared to pale yellow of wild type (LW-WT) (Fig. 1o). The non-native betaxanthins were stored in vacuole by observing the protoplasts extracted from carrot taproots (Fig. 1p). Expression of *Bx* in these three lines was confirmed by RT-qPCR (Fig. 1q). Betaxanthins content reached 15 mg/100 g FW in transgenic carrot taproots, which was considerable to yellow beetroots that naturally accumulated betaxanthins (Fig. 1r). The above data suggested that the 2A linker is a more efficient tool than NAL for metabolic engineering in horticultural plants.

The superior performance of the F2A linker over NAL10 can be attributed to their fundamental mechanistic differences. The F2A peptide operates via ‘ribosome-skipping’ where the ribosome continues translation of the downstream cistron without dissociation, leading to efficient co-translational separation of proteins [2]. In contrast, NAL10 facilitates downstream gene expression through ‘translation re-initiation’, a process that requires the ribosome to terminate, then re-assemble on the downstream start codon—a step inherently less efficient and more susceptible to interference [4]. The translation efficiency of the upstream cistron is not expected to differ between the two linkers, as F2A-mediated ribosome skipping and NAL10-mediated re-initiation both allow normal translation termination of the upstream gene. Our results, showing drastically higher expression of the second gene with F2A, are consistent with this mechanistic hierarchy and provide practical evidence for selecting ribosome-skipping linkers when high-level co-expression is critical. While this study focused on F2A, exploring other 2A variants (e.g. P2A, T2A) constitutes a logical next step to further optimize polycistronic expression in plants. In addition to NAL and 2A, the classic IRES element is another option. However, early studies in rice demonstrated that 2A was superior to IRES for driving downstream gene expression [5]. Therefore, 2A currently represents the optimal linker choice. It should be noted that linker performance can be context-dependent. Future studies incorporating diverse protein-coding sequences would be valuable for further generalizing this conclusion.

## Data Availability

The data sets supporting the conclusions of this article are included within the article.
